# Impact of ketogenic and fast-mimicking diet in gastrointestinal cancer treatment

**DOI:** 10.3389/fonc.2025.1677509

**Published:** 2025-11-07

**Authors:** Elisa Colombo, Margherita Righini, Vyshnavy Balendra, Konul Rustamli, Ornella Garrone, Margherita Ratti, Michele Ghidini

**Affiliations:** 1 National Federation of Orders of Biologists, Milan, Italy; 2 Postgraduate School of Medical Oncology, University of Parma, Parma, Italy; 3 Medical Oncology Unit, University Hospital of Parma, Parma, Italy; 4 Saint James School of Medicine, Park Ridge, IL, United States; 5 Laboratory of Molecular Pathology, Department of Health Sciences, University of Eastern Piedmont (UPO), Novara, Italy; 6 Oncology Unit, Fondazione IRCCS Ca’ Granda, Ospedale Maggiore Policlinico, Milan, Italy; 7 Oncology and Hematology Department, Piacenza General Hospital, Piacenza, Italy

**Keywords:** gastrointestinal (GI) cancers, ketogenic diet (KD), fasting-mimicking diet (FMD), β-hydroxybutyrate (β-HB), reactive oxygen species (ROS), insulin-like growth factor-1 (IGF-1), ketone bodies (KBs), oxidative stress (OxS)

## Abstract

Growing evidence suggests that both the ketogenic diet (KD) and the fast-mimicking diet (FMD) may have significant therapeutic effects in the treatment of gastrointestinal (GI) cancers. KD, characterized by a high fat intake and low carbohydrate intake, induces a state of ketosis that alters energy metabolism, reducing the availability of energy for cancer cells and slowing their growth. Similarly, FMT, which simulates the effects of fasting without requiring complete food abstention, has been studied for its potential to enhance immune response, reduce inflammation, and stimulate autophagy, contributing to the removal of damaged cells. Preclinical and clinical studies indicate that both dietary strategies may enhance the efficacy of chemotherapy while reducing the side effects associated with conventional treatments. Despite these promising findings, few studies have investigated the potential impact of these diets on anticancer treatment of gastrointestinal cancers, and further studies are necessary to better understand the biological mechanisms and to evaluate the safety and effectiveness of these strategies in broader clinical settings. With our review, we aim to analyze the available literature on KD and FMD and their role in the treatment of GI cancers.

## 1 Introduction

Gastrointestinal (GI) cancers represent a significant global health burden, accounting for a substantial proportion of cancer incidence and mortality worldwide. In the last years, the estimated global incidence of digestive system cancers reached approximately 4.9 million cases, representing 24.6% of all new cancer diagnoses (1, 2). Notably, there is a worrying trend towards an increase in the incidence of GI tumors among individuals under the age of 50 (3).

Nutrition has emerged as a critical factor influencing cancer progression and response to therapy. Dietary patterns can modulate systemic metabolism, inflammation, and the tumor microenvironment, thereby affecting tumor growth and treatment sensitivity (4, 5).

Among various dietary interventions, the ketogenic diet (KD) and fasting-mimicking diet (FMD) have garnered attention for their potential anticancer effects.

KD is a high-fat, low-carbohydrate diet that induces a metabolic state of ketosis, leading to reduced glucose availability and increased ketone body (KB) production (6, 7). Cancer cells rely heavily on glycolysis for energy production. By limiting glucose, KD may selectively starve cancer cells while providing an alternative energy source for normal cells. Particularly, KD can mimic the metabolic effects of fasting without significant calorie deprivation (6, 7). Preclinical studies have demonstrated that KD can inhibit tumor growth and enhance the efficacy of chemotherapy and radiotherapy (8). Moreover, in preclinical models, KD has been shown to modulate the tumor microenvironment, reduce inflammation, and improve responses to immune checkpoint inhibitors (9). Emerging data suggest that KD may potentiate anti-tumor responses by modifying insulin signaling, oxidative stress (OxS), and the tumor immune microenvironment (6–10).

Similarly, FMD involves periodic cycles of low-calorie, low-protein, and low-carbohydrate intake, mimicking the effects of fasting while providing essential nutrients (11). FMD, as reported, may enhance the efficacy of anticancer therapies by promoting tumor cell apoptosis, reducing systemic inflammation, and improving immune responses (11). Clinical trials have indicated that FMD is safe and feasible in cancer patients, with evidence suggesting improved treatment outcomes in various malignancies (11–13).

Recent studies have also highlighted the role of diet in modulating the gut microbiome, which plays a crucial role in immune regulation and cancer progression (14). Dietary interventions like KD and FMD can influence the composition and function of the gut microbiota (15), potentially impacting treatment responses and toxicity profiles.

Despite promising findings, the integration of KD and FMD into standard oncological care remains limited. Challenges include patient adherence, nutritional deficiencies, and the need for individualized dietary planning. Furthermore, GI tumors are highly heterogeneous; it is therefore mandatory to ask whether dietary interventions should be differentiated based on the type of tumor and the treatment.

This review aims to summarize current evidence on the impact of KD and FMD in the treatment of GI cancers, examining their mechanisms of action, clinical efficacy, and potential integration into multimodal cancer therapy.

## 2 Ketogenic and fast-mimicking diets

The conventional high-carbohydrate diet consists of glucose as the primary energy source. After digestion, through the aid of GLUT-4 transporters in muscle and adipose tissues, glucose enters the bloodstream (16). The increased glucose levels activate secretin to be released from pancreatic β-cells, thereby opening-up access to glucose and starting the process of glycolysis. Hexokinase, phosphofructokinase-1 (PFK-1), and pyruvate kinase are the essential enzymes involved in the conversion of glucose to form pyruvate (17). From here, pyruvate enters the mitochondria, and the enzyme pyruvate dehydrogenase (PDH) converts it to acetyl- CoA which is a substrate for the tricarboxylic acid (TCA) cycle and aids in oxidative phosphorylation to make adenosine triphosphate (ATP). An abundance of glucose from digestion is stored as glycogen or converted into fatty acids through lipogenesis, through the aid of the enzymes acetyl-CoA carboxylase (ACC) and fatty acid synthase (FAS) (18).

### 2.1 Ketogenic diet

**Figure 1:**
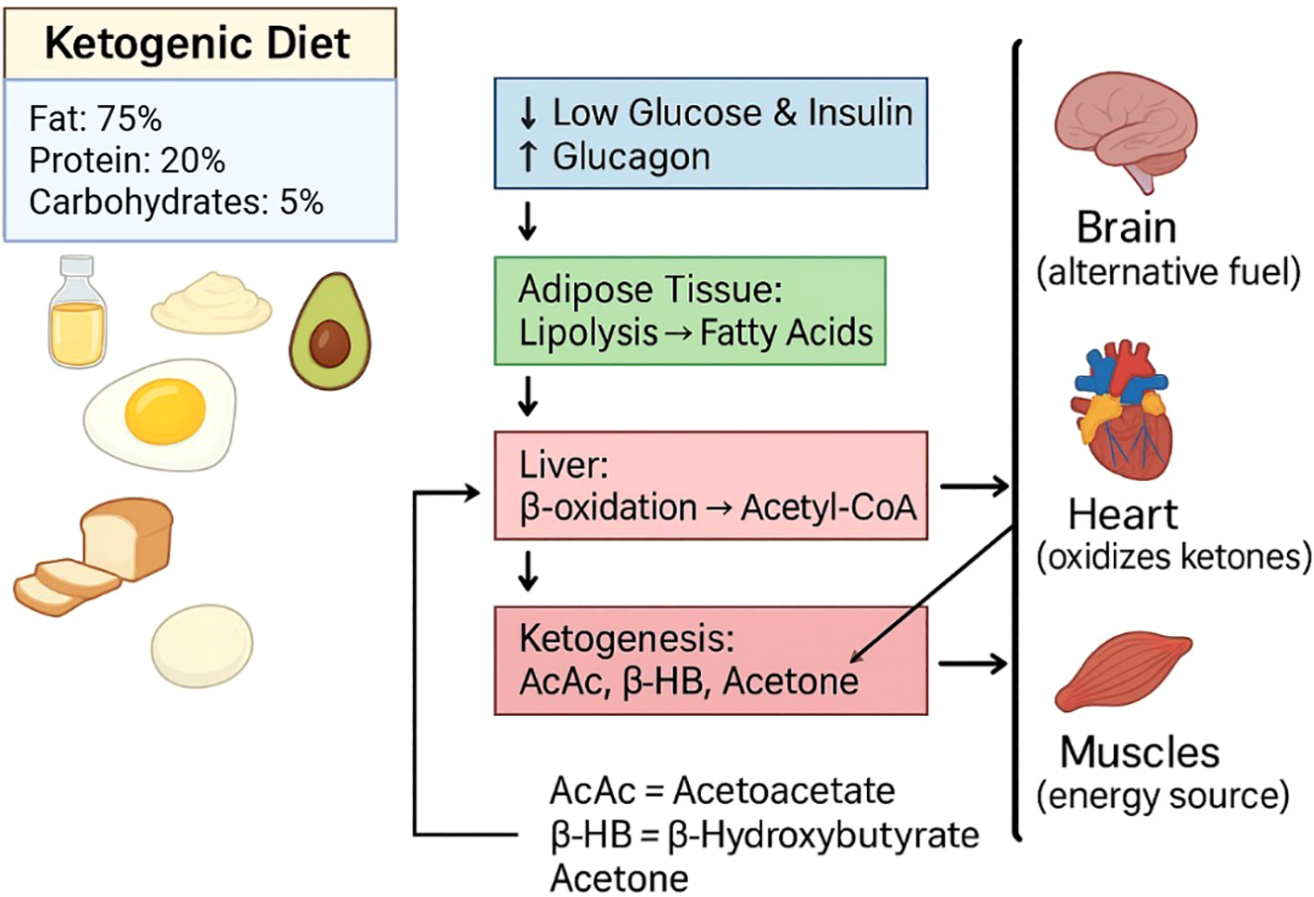
Ketogenic diet (KD). KD comprises high fat (~75%), moderate protein (~20%), and very low carbohydrates (~5%). Low carbohydrate intake reduces glucose and insulin levels while increasing glucagon, which directs the process of lipolysis in adipose tissue. Fatty acids released undergo β-oxidation in the liver, producing Acetyl-CoA. Excess Acetyl-CoA enters the ketogenesis pathway to form ketone bodies: acetoacetate (AcAc), β hydroxybutyrate (β-HB), and acetone. These ketones are released into circulation and serve as alternative fuels for the brain, heart, and skeletal muscle, during periods of carbohydrate restriction.

KD is a low-carbohydrate high-fat diet that is fueled by fatty acids and KBs. (**Figure 1**).

The decreased levels of insulin, and increased levels of glucagon activates the hormone-sensitive lipase (HSL) involved in lipolysis and secretes non-esterified fatty acids (NEFAs) from fat cells (19). NEFAs are later transported to the liver and activated by acyl-CoA synthetase. Through the carnitine shuttle and enzymes such as carnitine palmitoyl-transferase I and II (CPT-I and CPT-II), NEFAS enter the mitochondria (20).

β-oxidation takes place in the mitochondria creating acetyl-CoA. Ketogenesis occurs where KB are made from acetyl-CoA from HMG-CoA synthase 2 and HMG-CoA lyase. The end products are AcAc, which are further reduced to β-hydroxybutyrate (β-HB) and acetoacetate (AcAc) (21).

During long periods of fasting, KBs serve as energy stores for the brain, heart, and skeletal muscles. Through monocarboxylate transporters (MCTs), β-HB and AcAc can be converted to acetyl-CoA by the enzymes succinyl-CoA:3-ketoacid CoA transferase and thiolase to enter the TCA cycle and provide energy (22). This specific form of metabolism results in decreased reactive oxygen species (ROS). Ketone metabolism produces less ROS and subsequently reduces OxS and improves cellular vitality (23). KD increases fat oxidation and ketogenesis, decreases the utilization of glycolytic enzymes, which creates an adverse biochemical environment for pathologies such as type 2 diabetes mellitus and cancer to sustain themselves, in comparison to the high-carb diet (24).

### 2.2 Fasting-mimicking diet

FMD consists of reduced calories, protein, carbohydrate while upholding a high-unsaturated fat content. In comparison to a regular diet, with increased levels of insulin and insulin-like growth factor-1 (IGF-1) creating an anabolic state, the FMD creates a catabolic metabolic state with reduced glucose, insulin, and IGF-1 levels, and increased glucagon, adiponectin, and KBs (25). These changes in hormones activate lipolysis and autophagy. Consequently, the decreased amount of IGF-1 inhibits the PI3K-Akt-mTOR pathway, thereby decreasing cell cycle activation and cellular oxidative damage (26).

Due to the low carbohydrate intake, FMD has negligible hepatic glycogen stores and biochemically energy metabolism is directed at fat oxidation and ketogenesis. Fatty acids from adipose tissue travel to the liver and activate β-oxidation creating acetyl-CoA which activates KB synthesis. β-HB and AcAc, ketones, are directed at tissues to make ATP. Due to the reduced protein intake in FMD, mTOR pathway is suppressed which increases autophagy and increases the removal of misfolded proteins and damaged cells which inadvertently improves organ function and longevity (27).

## 3 Biological and molecular implications of KD and FMD on cancer treatment

Cancer is a disease characterized by mutations and a selection process that occurs within an organism, leading to the emergence of a cell population that thrives at the expense of the organism (28). Activated oncogenes and mutant tumor suppressors in tumor cells cause a fundamental change in energy metabolism that facilitates cell growth and proliferation (28). This metabolic change can lead to mitochondrial alterations and, potentially, the inability of tumor cells to fully metabolize KBs as a source of energy and useful metabolic intermediates (29). This has led to the hypothesis that, during ketosis, normal healthy cells would continue to generate energy from KBs, while tumor cells would be unable to metabolize these compounds (30). Tumor cells show increased glucose uptake and lactate production compared to normal cells, indicating elevated glycolytic activity and increased energy metabolism (31).

**Figure 2:**
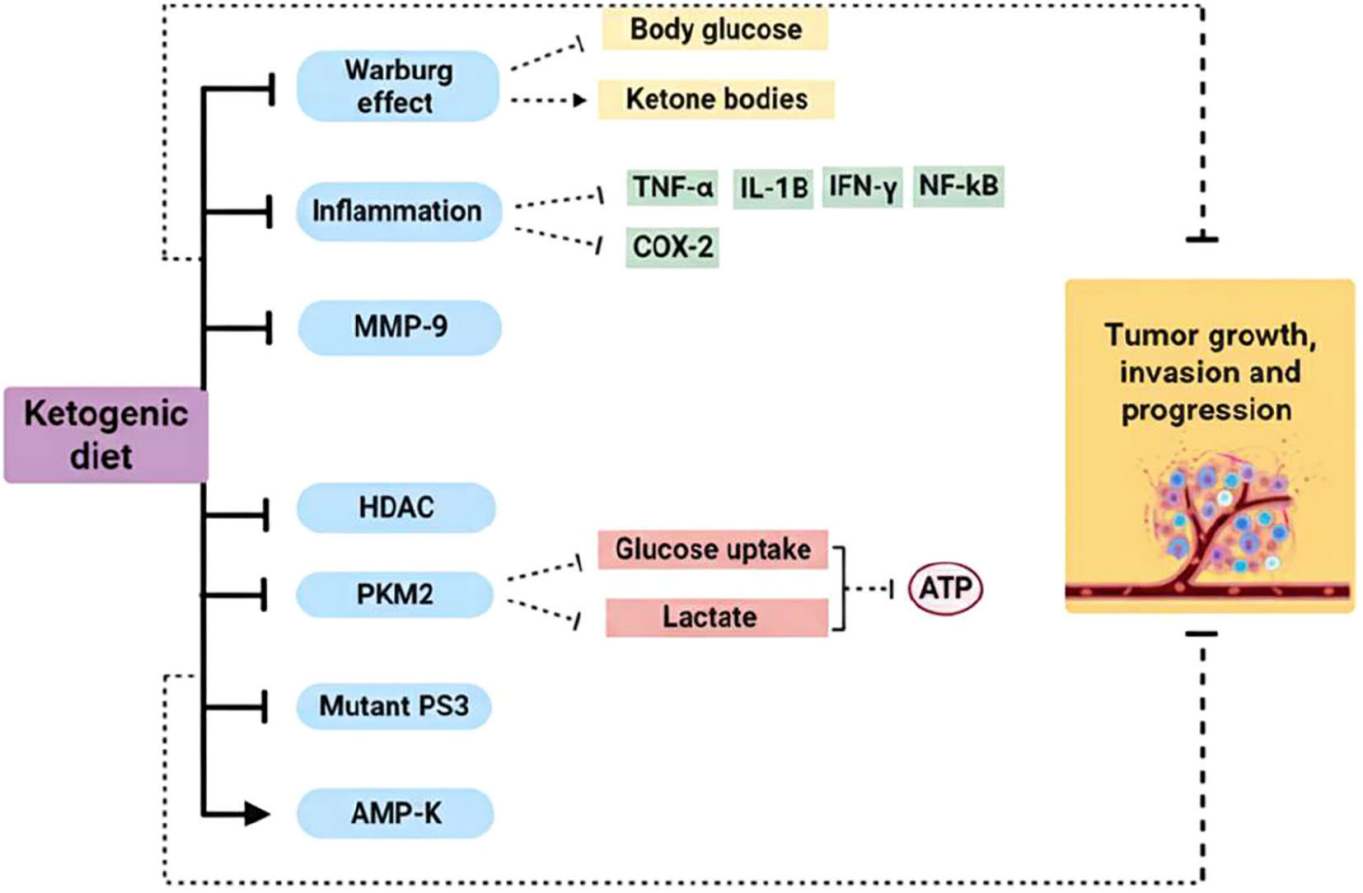
Ketogenic diet mechanism of action as a cancer therapy. Cancer cells are characterized by a metabolic alteration called the Warburg effect, which involves increased aerobic glycolysis to produce ATP. KD reduces glucose levels in the body and, conversely, increases levels of ketone bodies, which cannot be used by tumor cells as substrates for ATP production. KD use has shown reduced expression of pro-inflammatory cytokines and cyclooxygenase 2. These anti-inflammatory effects may improve the response to cancer treatment and prevention. Furthermore, this type of diet can reduce the expression of MMP-9 and HDAC in several types of cancer, as well as being implicated in the reduction of mutant P53 expression. With KD, the expression of PKM2 is attenuated, thus reducing glucose uptake and lactate production in the tumor cell, while AMPK is activated, which plays a key role in cancer prevention. (→), activation; (┬), inhibition. TNF-α, tumor necrosis factor-α; IFN-γ, interferon-γ; IL-1β, interleukin-1β; NF-κB, nuclear factor-κB; COX-2, cyclooxygenase-2; MMP-9, matrix metalloproteinases-9; HDAC, histone deacetylase; PKM2, pyruvate kinase M2; mutant p53, mutant nuclear transcription factor; AMP-K, AMP-activated protein kinase.

This phenomenon, where glycolysis is mainly used instead of the normal aerobic cycle of cells, is called the Warburg effect (32). Increased glycolysis, decreased tricarboxylic acid cycle activity and oxidative phosphorylation are found at the beginning of tumorigenesis, which is characteristic of tumors (33), (**Figure 2**).

### 3.1 Metabolic effect of KD and β-HB

In this context, KD can influence tumor cell growth by lowering insulin and insulin-like growth factor-1 (IGF-1) levels, thus reducing one of the most common oncogenic pathways such as PI3K-Akt-mTOR implicated in tumor cell proliferation and tumor growth (34, 35).

Particularly, KBs produced by KD participate in various cellular processes as signaling molecules, regulate inflammation and OxS. The latter is recognized to contribute to a wide range of acute and chronic diseases, including cancer, cardiovascular disease and lung disease (36). Alterations in mitochondrial structures and functions in tumor cells result in increased generation of ROS compared to healthy cells. In addition, KD increases the concentration of ROS in tumor cells, which become highly vulnerable to OxS and can be damaged (37).

Related to the development of neoplastic diseases is also inflammation (38) which can contribute to the initiation, promotion and metastasis of cancer (39). It has been revealed that approximately 25% of cancer cases are related to chronic inflammation (40). When it comes to modulating the inflammatory microenvironment for cancer therapy, inflammatory cytokines are the most efficient targets. Antitumor therapy combined with KD has shown reduced expression of COX-2 and pro-inflammatory cytokines, such as tumor necrosis factor-α (TNF-α), interferon-γ (IFN-γ) and interleukins (including IL-1β) through the inhibition of NF-κB (41). This decreases the inflammatory picture closely linked to tumor progression and resistance to cancer treatment (42).

Furthermore, the most abundant ketogenic body, β-HB inhibits inflammatory responses and immune cell function by binding to and activating the hydroxycarboxylic acid receptor 2 (HCAR2) or directly modulating some intracellular signaling pathways (43, 44).

β-HB reduces colonic crypt cell proliferation and inhibits intestinal tumor growth through HCAR2 activation and Hopx transcriptional regulator induction, thereby altering gene expression and inhibiting tumor cell proliferation. Similarly, the antitumor properties of KD are correlated with the downregulation of pyruvate kinase M2 (PKM2) expression levels, a key enzyme that limits the rate of glycolysis (45).

Furthermore, the following studies have demonstrated an overexpression of matrix metalloproteinases (MMP-1, 2, 3, 7, 9 and 13) in human colorectal cancer (46) and gastric cancer (47). Several MMPs play a role in cancer progression, migration, invasion, metastasis and angiogenesis (48). The use of KD, in combination with other conventional anticancer therapies, has been shown to provide a significant reduction in MMP-9 expression in several cancer types (49).

Numerous studies have demonstrated the relationship between KD, KBs and the suppression of histone deacetylases (HDACs) expression in human tumors (48). These enzymes play a central role in the regulation of several cellular properties that are closely linked to cancer development and progression (49). Accordingly, HDAC inhibitors have been shown to induce specific changes in gene expression and influence growth arrest, differentiation, cytotoxicity and induction of apoptosis (50).

Cell proliferation and apoptosis are also controlled by the nuclear transcription factor p53 (51). In most tumors, p53 is mutated with prolonged half-life and resistance to therapy (52). KD has been shown to block the activity of mutant p53 or silences its expression during malignant initiation and progression (48, 53).

### 3.2 Metabolic effect of FMD

**Figure 3:**
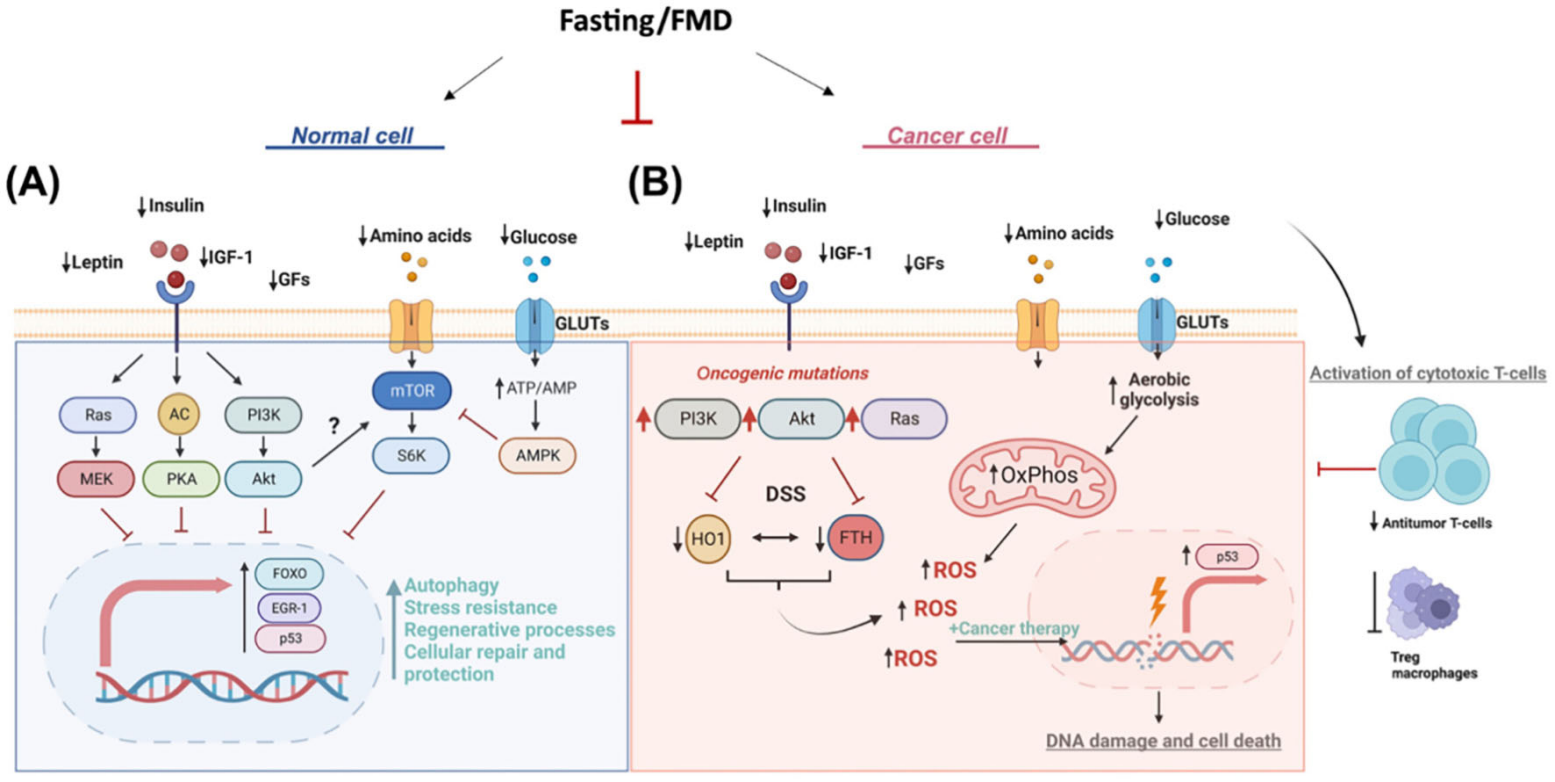
Multiple effects of FMD in cancer treatment. **(A)** Normal cell. During fasting, reduced levels of leptin, insulin, IGF-1, and growth factors (GFs) inhibit key proliferative and anabolic signaling pathways, including Ras/MEK, AC/PKA, and PI3K/Akt. Decreased amino acid and glucose availability leads to reduced activation of mTOR/S6K and an increase in the ATP/AMP ratio, which in turn activates AMPK. These signals converge on the activation of the transcription factors FOXO, EGR-1, and p53, which promote autophagy, stress resistance, regenerative processes, repair, and cellular protection. **(B)** Tumor cell. The same fasting conditions reduce leptin, insulin, IGF-1, GFs, amino acids, and glucose, but the presence of oncogenic mutations in the PI3K/Akt and Ras pathways alters the metabolic and signaling response. Furthermore, fasting stress (DSS) induces an increase in HO1 and FTH, resulting in increased oxidative phosphorylation (OxPhos) and aerobic glycolysis. These processes lead to the production of reactive oxygen species (ROS), amplified by anticancer treatments, which causes DNA damage and cell death. Fasting promotes the activation of cytotoxic T cells, reducing the activity of immunosuppressive Tregs and macrophages, contributing to a strengthened antitumor immune response.

Another dietary intervention that has been studied in cancer patients to improve quality of life, regulate tumor growth, and improve tolerance to side effects of anticancer treatments is FMD. Fasting may benefit cancer patients through several mechanisms. One of these is “differential stress resistance, “ in which fasting protects healthy cells from the toxic effects of chemotherapy by promoting a protective low-growth mode, while tumor cells remain vulnerable due to their rapid proliferation. This selective protection reduces DNA damage and side effects in healthy tissues (54). Furthermore, fasting improves immune function by stimulating autophagy and facilitating the regeneration of immune cells, strengthening the body’s response to infections, and improving the efficacy of anticancer treatments by reducing tumor immunosuppression (55). Furthermore, fasting lowers insulin, glucose, and inflammatory markers, thus slowing tumor progression with positive outcomes on survival and quality of life (56) (**Figure 3**).

The ability of FMD to sensitize tumors to treatment has been attributed to the downregulation of IGF-1 and heme oxygenase 1 (HO-1) (57), an antioxidant protein with antiapoptotic and cytoprotective effects through its catabolites in addition to the clearance of toxic intracellular heme. Evidence suggests that HO-1 promotes tumor progression and plays an immunomodulatory role that influences tumor-associated immune infiltrate, including Treg cells (58). FMD is able to modulate systemic metabolism and antitumor immunity by reducing the frequency of immunosuppressive regulatory T cells (Treg) in peripheral blood. In combination with chemotherapy, FMD enhanced immunosurveillance and antitumor immune cytotoxicity mediated by CD8+ T cells (59).

It is also important to consider the activity of fasting and/or fasting-mimicking conditions on the metabolism and autophagy flux of immune cells, as both significantly influence the outcome of immune responses (60). Furthermore, fasting and/or fasting-mimicking conditions can alter the composition and metabolism of the microbiota (61).

## 4 Clinical studies in GI cancers

Given the marked heterogeneity of GI cancers, dietary interventions such as KD and FMD must be analyzed within the context of individual tumor subtypes. Current evidence suggests that both strategies are generally safe and feasible, with potential benefits ranging from improved body composition and metabolic profiles to enhanced synergy with systemic therapies and immunotherapy. While clinical data remain preliminary and often derived from small or retrospective studies, early findings support the hypothesis that metabolic modulation may represent a valuable adjunctive approach in GI oncology. The following sections review available evidence, organized by cancer subtype (**Table 1**).

**Table 1:** Studies evaluating KD and FMD in GI cancers.

Study (author, year)	Cancer type	Study design/N	Diet intervention	Treatment combination	Key outcomes
Zahra et al. (2017) (62)	NSCLC and PC	Phase I Trials; N = 9	KD	CTRT	Radiosensitization observed preclinically; poor dietary adherence limits clinical feasibility
Hagihara et al. (2020) (63)	Advanced Cancers (CRC and PC)	Prospective case series; N = 55	KD (gradual carb increase + ESPEN-based support)	Standard therapies	Median OS: 32.2 months; good tolerability; improved ketone levels, glucose, insulin, and CRP
Jameson et al. (2024) (65)	Advanced PC	Randomized Phase II; N = 32	MSKD	first-line triplet CT (gemcitabine, cisplatin, nab-paclitaxel)	Feasible and safe; promising for combination with chemotherapy
Iyikesici (2020), (66)	Metastatic PC	Retrospective cohort; N = 25	KD	Gemcitabine-based or FOLFIRINOX + Hyperthermia + HBOT	Extended OS; survival superior to historical GEM/FOLFIRINOX cohorts
Klement et al. (2021) (67)	RC	Controlled clinical trial; N = 49	KD	neoadjuvant CTRT	Maintained lean mass; favorable metabolic shifts
Klement et al. (2022) (68)	RC	Prospective study; N = 49	KD	neoadjuvant CTRT	Improved QoL and metabolic markers
Furukawa et al. (2019) (69)	Stage IV CRC	Retrospective study; N = 10	KD	CT	Improved disease control rate; feasible
Sims et al. (2023) (70)	stage III CRC	Case report + literature review	KD	CT-IT	feasibility and tolerability of integrating a ketogenic diet with both chemotherapy and immunotherapy
Vernieri et al. (2022) (11)	Mixed (includes GI cancers)	Phase I/II Monocentric; N = 101	FMD cycles	Standard therapies	High adherence; metabolic benefits (↓glucose, insulin, IGF-1); immune activation
Ligorio et al. (2022) (73)	Mixed (includes CRC, PC)	Sub-analysis of Phase Ib; N = 101	FMD cycles	Standard therapies	supports FMD synergy with systemic therapy and immunity
Iyikesici (2020), (74)	Advanced gastric cancer (stage III–IV)	Retrospective cohort; N = 37	KD with caloric restriction	CT+ Hyperthermia + HBOT	Improved OS and PFS; well-tolerated

CRC, colorectal cancer; CT, chemotherapy; CTRT, chemoradiotherapy; CT-IT, chemo-immunotherapy; CRP, C reactive protein; HBOT, hyperbaric oxygen therapy; KD, ketogenic diet; FMD, fast-mimicking diet; IGF-1, insulin growth factor-1; MSKD, medically supervised ketogenic diet; N, number; NSCLC, non-small cell lung cancer; RC, rectal cancer; PC, pancreatic cancer; PFS, progression-free survival; OS, overall survival.

### 4.1 Pancreatic cancer

Pancreatic cancer (PC) has been one of the most extensively investigated GI malignancies in relation to KD. Early feasibility studies highlighted both the promise and the challenges of implementing strict dietary regimens. Zahra et al. (62) conducted two phase I trials evaluating KD in combination with chemoradiotherapy in patients with locally advanced non-small cell lung cancer and PC. While preclinical evidence suggested strong radiosensitizing and oxidative stress–inducing effects, clinical feasibility was limited. Of the two PC patients enrolled, only one was able to complete the intervention, with the other withdrawn due to dose-limiting toxicity. These findings underscored the difficulties of implementing highly restrictive KDs during intensive therapy, highlighting the need for more patient-adapted approaches.

Subsequent studies have provided more encouraging results. Hagihara et al. (63) tested a progressive KD protocol in 55 patients with advanced malignancies, including PC, gradually liberalizing carbohydrate intake from 10 to 30 g/day. Protein and caloric intake were adjusted based on ESPEN guidelines for cancer malnutrition.

These guidelines state that the nutritional treatment of cancer patients should rely on an estimation of the total energy expenditure (TEE), given by the sum of the resting (REE) and activity-associated energy expenditure. In case REE or TEE could not be measured, 25–30 kcal/kg/day with 1.2-1.5 g/protein/kg/day could be considered as proper ranges to maintain or restore lean body mass (64).

The progressive KD protocol demonstrated both safety and feasibility, with patients achieving sustained nutritional ketosis and improvements in metabolic markers such as serum ketone levels, fasting glucose, and C-reactive protein. Importantly, in the pancreatic cancer subgroup, a median overall survival (OS) of 10.7 months was observed, exceeding historical benchmarks for gemcitabine-based regimens (8.5–10.1 months) (63).

In the randomized phase II trial conducted by Jameson et al. (65), a medically supervised KD (MSKD) was combined with intensive chemotherapy (gemcitabine, cisplatin, nab-paclitaxel) in patients with metastatic PC. Compliance was remarkably high (94%), with only mild grade 1–2 adverse events and no severe metabolic complications. These findings suggest that KD can be integrated even into intensive systemic regimens without compromising safety.

Retrospective analyses have provided additional support. Iyikesici et al. (66) tested a multimodal metabolic protocol combining KD, metabolically supported chemotherapy, local hyperthermia, and hyperbaric oxygen therapy (HBOT). In 25 metastatic PC patients, this regimen yielded a median OS of 15.8 months and progression-free survival (PFS) of 12.9 months, surpassing expected outcomes with standard chemotherapy. Importantly, no severe adverse events attributable to KD or other metabolic components were observed.

Overall, clinical evidence in PC suggests that KD is safe, metabolically beneficial, and may synergize with chemotherapy, although patient adherence and protocol design remain critical determinants of feasibility.

### 4.2 Colorectal cancer

Colorectal cancer (CRC) has also been a major focus of KD and FMD research. In the KETOCOMP study (67, 68), 49 rectal cancer patients undergoing neoadjuvant radiotherapy were randomized to either KD (<50 g carbohydrates/day) or standard diet. KD resulted in significant fat mass reduction while preserving skeletal muscle mass, with no signs of sarcopenia. Patients also reported improved quality of life, particularly in emotional and social functioning, and lower incidence of treatment-related toxicities. These findings indicate that KD can modulate body composition and improve tolerance to radiotherapy in CRC patients.

Additional evidence of clinical benefit has been reported in advanced disease. Furukawa et al. (69) investigated a modified medium-chain triglyceride KD in 10 patients with stage IV CRC. The KD-chemotherapy group demonstrated an objective response rate (ORR) of 60% compared to 21% in controls, and a disease control rate (DCR) of 70% versus 64%. Notably, five patients in the KD arm achieved sufficient tumor regression to undergo conversion surgery, a rare outcome in metastatic CRC. Although the overall survival difference did not reach statistical significance, responders in the KD group achieved a median OS of 50 months, compared to 32.5 months in the chemotherapy-only group and 23.0 months in non-responders, suggesting a clinically meaningful trend.

KD may also have potential synergy with immunotherapy. A case report by Sims et al. (70) described a metastatic CRC patient who maintained disease stabilization for over a year under KD combined with trifluridine/tipiracil, bevacizumab, and later checkpoint inhibitors (ipilimumab/nivolumab). The patient maintained nutritional ketosis throughout, with preserved quality of life. This case aligns with preclinical data showing that KD can reprogram immune metabolism and reduce tumor-associated inflammation.

Beyond KD, FMD has shown mechanistic and translational promise in CRC. Preclinical studies demonstrated that FMD delayed tumor progression by reducing IgA-producing B cells and restoring CD8^+^ T-cell activity, thereby shifting the tumor microenvironment toward immunostimulation (71). Preliminary translational evidence confirmed similar immune-metabolic modulation in CRC patients. Furthermore, the combination of FMD with vitamin C in KRAS-mutant CRC models enhanced cytotoxicity through increased ROS and iron accumulation (72), suggesting a novel combinatorial therapeutic approach.

Clinical evidence from Vernieri et al. (11) further supported the feasibility of cyclic FMD in patients with solid tumors, including CRC. The protocol, consisting of 5-day plant-based low-calorie cycles every 21–28 days, was well tolerated, with high adherence and only mild adverse events. Significant reductions in fasting glucose, insulin, and IGF-1 were observed, alongside evidence of immune activation, including increased CD8^+^T cells and interferon-γ–related tumor signatures. A sub-analysis by Ligorio (73) of the same trial reported complete and durable responses in a subset of advanced cancer patients, including metastatic CRC, when FMD was combined with systemic therapy.

Taken together, these findings suggest that both KD and FMD hold therapeutic potential in CRC, with KD demonstrating improved body composition and possible synergy with chemotherapy and immunotherapy, while FMD appears particularly promising in reprogramming tumor metabolism and immunity.

### 4.3 Gastric cancer

Compared with pancreatic and colorectal malignancies, clinical evidence for KD in gastric cancer is more limited but nonetheless noteworthy. Iyikesici et al. (74) reported outcomes from 24 advanced gastric cancer patients, the majority of whom had metastatic disease, treated with a multimodal metabolic approach including KD, metabolically supported chemotherapy, hyperthermia, and HBOT. Remarkably, the overall response rate was 100%, with 88% complete responses confirmed by histology and PET-CT. Mean OS was 39.5 months, and PFS reached 36.5 months, despite many patients presenting with poor baseline performance status (ECOG ≥2). No severe adverse events were reported, reinforcing the safety of the multimodal approach.

Although these results are striking, the multimodal nature of the intervention complicates the attribution of efficacy specifically to KD. Nonetheless, they highlight the potential of metabolic interventions to enhance therapeutic responses in gastric cancer, warranting prospective randomized studies.

### 4.4 Discussion and ongoing studies

Emerging clinical evidence supports the therapeutic potential of dietary interventions—particularly KD and FMD—as adjuncts to conventional treatments in gastrointestinal malignancies. Their ability to influence tumor progression through both intrinsic metabolic stress and extrinsic immune modulation positions them as compelling candidates for integration into oncologic care.

Across the clinical studies reviewed, encompassing diverse GI malignancies, KD, often combined with standard chemotherapy, radiotherapy or other treatments demonstrated favorable safety profiles and promising therapeutic effects. Several investigations, particularly those involving metabolically supported chemotherapy protocols in advanced gastric and PC reported prolonged survival outcomes when KD was administered alongside systemic treatment. Notably, some studies revealed that inducing metabolic stress through KD and adjunctive therapies, including hyperthermia and hyperbaric oxygen, may selectively sensitize tumor cells to cytotoxic agents while protecting normal tissues. In PC and colorectal cancer settings, early-phase trials and observational reports highlighted the feasibility and tolerability of ketogenic interventions, including medically supervised regimens. These approaches were associated with improved metabolic parameters (e.g., reduced insulin and glucose levels), maintenance of body composition, and, in some instances, improved quality of life during chemoradiotherapy. Furthermore, mechanistic insights suggest that KD may modulate tumor metabolism and immune responses, thereby contributing to a more therapeutically responsive tumor microenvironment.

Complementing these findings, early-phase clinical and preclinical studies on FMD have highlighted a parallel role in modulating systemic metabolism and tumor immunobiology, while being both feasible and safe. These results provide a strong rationale for ongoing and future randomized trials evaluating the efficacy of FMD as an adjunct to standard chemotherapy, targeted therapies, and immunotherapies.

Among ongoing studies or trials with unpublished results testing FMD, the randomized-controlled CHEMOFAST trial enrolled 11 patients with CRC treated with chemotherapy. The experimental arm followed a short-term FMD for 44–48 hours, starting 24 hours before chemotherapy, while the control arm received standard dietary indications. The trial had a primary endpoint of safety, evaluating changes in the Common Terminology Criteria for Adverse Events (CTCAE) 5.0 toxicity table score between baseline and after three weeks of treatment. The study has completed recruitment, and results have not been published yet (NCT04247464). The randomized-controlled Chinese FCRC22 trial is recruiting stage III CRC patients receiving adjuvant chemotherapy. The experimental arm follows a cyclic FMD consisting of a 5-day regimen (day 1 supplies 1000 kcal, days 2–5 provide 800 kcal). At least 4 FMD cycles are planned after surgery. On the contrary, the standard arm follows a regular diet, with a total of 602 patients expected to be included and a primary endpoint of disease-free survival at 3 years (NCT05384444). A phase Ib trial is testing the safety, side effects, and effectiveness of the monoclonal antibodies botensilimab and balstilimab in combination with FMD and high dose vitamin C in treating patients with *KRAS* mutant metastatic CRC. FMD is tested together with these monoclonal antibodies in this setting because *KRAS* mutant cells have been found to be more sensitive to vitamin C induced growth suppression in the presence of low sugar (glucose) (NCT06336902).

KD is being tested in locally advanced rectal cancer undergoing neoadjuvant radiotherapy in the ongoing randomized-controlled KOMPARC study. The interventional group will be prescribed a KD therapy plan characterized by the following composition: carbohydrates < 30g/day, 1.2g-1.5g protein/kg/day and lipids > 65%. Differently, the standard group will be prescribed a regular nutrition plan according to ESPEN guidelines. Primary endpoint is the evaluation of the compliance with KD compared to standard diet (NCT05938322). Similarly, a randomized-controlled phase II study evaluated the 3-year PFS in patients with metastatic PC on triplet therapy (nab-paclitaxel + gemcitabine + cisplatin) while on KD (experimental) or non-KD (standard arm). KD consisted in carbohydrates < 30g/day and a daily protein intake targeted to 1.5 g/kg/day. The study has recently completed its accrual.

### 4.5 Conclusions

Both KD and FMD may have a significant therapeutic impact in the treatment of GI cancers. Preclinical and clinical studies indicate that both dietary strategies may enhance the efficacy of chemotherapy while reducing the side effects associated with conventional treatments.

Despite these encouraging findings, current evidence remains preliminary. Most studies are limited by small sample sizes, heterogeneous protocols, and the lack of randomized controlled designs. Nevertheless, the consistent signal of metabolic and clinical benefits observed across tumor types provides a compelling rationale for further investigation. Another important limitation relates to the fragility of this patient group and the difficulty of adhering to such a restrictive diet. Compliance with the diet and daily food intake may be affected by side effects such as nausea, loss of appetite, dysphagia, and dysgeusia. While further randomized controlled trials are needed to validate these results and explore long-term oncologic outcomes, these studies provide encouraging evidence for the integration of metabolic strategies into multimodal cancer care.
